# Retraction: Deep learning for autism diagnosis and facial analysis in children

**DOI:** 10.3389/fncom.2023.1215827

**Published:** 2023-05-16

**Authors:** Frontiers Editorial Office

The journal and Chief Editors retract the 20 January 2022 article cited above.

Following publication, concerns were raised regarding the quality of the article and the language of the presentation. An investigation was conducted in accordance with Frontiers' policies.

A post-publication assessment by an expert in the field found that the complaints were valid and that the study design is not adequate to justify the conclusions drawn by the authors. The article does not meet the standards of editorial and scientific soundness for Frontiers in Computational Neuroscience; therefore, it has been retracted.

This retraction was approved by the Chief Editors of Frontiers in Computational Neuroscience and the Chief Executive Editor of Frontiers. The authors did not agree to this retraction.

